# Pre-emptive Analgesic Efficacy of Single-Dose Transdermal Ketoprofen and Diclofenac Patches in Post-operative Pain Management Following Open Treatment of Mandibular Fractures: A Randomized Controlled Study

**DOI:** 10.7759/cureus.27982

**Published:** 2022-08-13

**Authors:** Akhil A Sharma, Anendd A Jadhav, Nitin D Bhola, Aishwarya A Gupta, Chetan S Gupta

**Affiliations:** 1 Oral and Maxillofacial Surgery, Sharad Pawar Dental College, Datta Meghe Institute of Medical Sciences, Wardha, IND

**Keywords:** analgesia, transdermal patch, ketoprofen, diclofenac sodium, mandibular fracture, pain

## Abstract

Introduction: The present study was deliberated to assess the pre-emptive analgesic efficacy of diclofenac sodium and ketoprofen transdermal patches following open treatment of mandibular fractures.

Methods: The present prospective, triple-blind, randomized controlled clinical study was carried out on 50 male patients with a mean age of 30-31 years having bifocal mandibular fractures. The subjects were assigned 1:1 to two groups; group K - ketoprofen group and group D - diclofenac sodium group. Patches were applied according to the group allocation one hour before induction. In the immediate post-operative (PO) phase, pain intensity was recorded using a 10-point Visual analog Scale at 2, 4, 8, 12, and 24 hourly. Statistical analysis was performed using descriptive and inferential statistics using SPSS 27.0 version (IBM SPSS, Armonk, NY) and GraphPad Prism 7.0 version (GraphPad Software, Inc., La Jolla, CA) and p<0.05 is considered a level of significance.

Results : The present study demonstrated a statistical difference in mean pain intensity among both groups, with lower pain scores at all time intervals and fewer rescue analgesic consumption in the ketoprofen group.

Conclusion: The ketoprofen transdermal patch was found to be superior in comparison to the diclofenac patch in terms of providing optimal post-operative analgesia with a reduced requirement for post-operative rescue analgesics and minimal adverse events.

## Introduction

The mandible, being a prominently placed bone in the maxillofacial skeleton, is vulnerable to fractures. Wherever indicated, these are optimally treated using internal fixation to restore premorbid occlusion, pain-free jaw movements, mastication, speech, and establishment of facial symmetry. Open treatment of these fractures mandates considerable reflection of mucoperiosteal tissues, stripping of muscles, and mobilization of fracture fragments. This results in the triggering of the secretion of a cascade of mediators of pain and inflammation, which translates into the pain of greater intensity. Adequate post-operative (PO) pain control is a critical tenet of perioperative care and an indicator of therapeutic success of treatment, recovery, and functional outcomes. Failure to provide adequate analgesia in the acute PO phase might result in physical and psychological discomfort for the patient, longer hospital stays, protracted course of recovery, escalating cost of treatment, and adversely impacting PO quality of life [1,2].

Peripherally acting non-steroidal anti-inflammatory drugs (NSAIDs) and centrally acting opioid analgesics are either used as a sole modality or as part of the multimodal strategy mainstay of the therapy to control moderate to severe intensity pain in acute PO setting. Systemic routes of administration are routinely associated with less compliance and an increased frequency of side effects like acid peptic disorders, dyspepsia, gastroduodenal ulcers, gastrointestinal bleeding, hypertension, and edoema [3,4,5]. To avoid these gastrointestinal complications, a transdermal drug delivery system (TDDS) was introduced, which generated special interest.

Evidence-based procedure-specific post-operative analgesic guidelines are lacking in maxillofacial surgery. One of the most popular NSAIDs used for analgesia is diclofenac sodium. It works by blocking the cyclooxygenase (COX) enzyme, which inhibits prostaglandin (PG) synthesis and thereby exhibits analgesic and antipyretic properties. Ketoprofen is another drug, which apart from suppressing COX and lipoxygenase (LOX) pathway also instigates central and peripheral desensitization. Unlike other NSAIDs, ketoprofen acts at both the peripheral and central levels by inhibiting central prostaglandin production (brain COX and nitric oxide synthase), thus exhibiting better efficacy compared to diclofenac [2,3,6-8].

TDDS exhibits several advantages over systemic routes. It is minimally invasive with greater ease of administration, circumvents the first pass metabolism, helps maintain uniform drug concentration over a prolonged period, and helps to preclude patient variability and helps in individuals with trypanophobia [5,6]. The comparative effectiveness of ketoprofen vs diclofenac in TDDS was evaluated with consistency in dentoalveolar surgery, impacted third molar surgery [9], and bi-jaw orthognathic surgery [10]. Its effectiveness in mandibular fractures is a matter of investigation. In light of the above background, the present study was designed to assess the profoundness of transdermal ketoprofen patch over diclofenac sodium in terms of achieving profound post-operative analgesia and safety in subjects with mandibular fractures following open reduction and internal fixation. The primary outcome variables were the duration of analgesia, the mean VAS score, and the time to take rescue analgesia. Any adverse reactions were also noted.

As there is a paucity of literature comparing ketoprofen and diclofenac transdermal patches in treating post-operative pain following open reduction of the mandibular fracture, the following study was conducted with the hypothesis that pre-emptive ketoprofen transdermal patches are more efficacious than diclofenac patches in reducing post-operative pain following open treatment of mandibular fractures. Post-operative analgesia, pain severity, and consumption of rescue analgesics were considered the outcome variables of the study.

## Materials and methods

**Figure 1:**
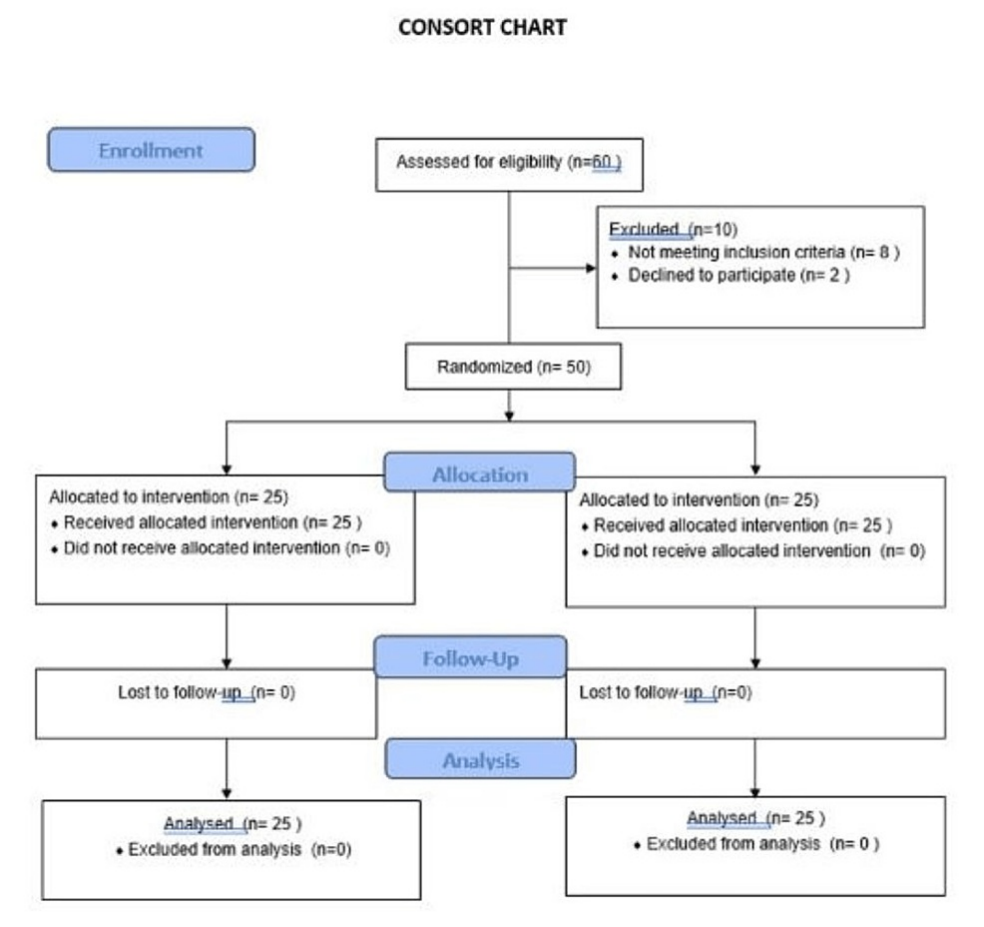
Consort chart

The present prospective, randomized-controlled clinical study was deliberated as a prospective randomized, parallel-arm, clinical study on 50 consecutive patients. The study was conducted between September 2020 and September 2021 in the oral and maxillofacial surgery departments. The study was performed in accordance with the consolidated standards of reporting trials (CONSORT) [11] (Figure 1).

The sample size for the present study with a 95% confidence interval and 80% power of the study was derived using the formula:

*N* = (*Zɑ*/2 + *Zβ*) 2 × 2 × *σ*2/*d*2

where “*Zɑ*/2” is the critical value of the normal distribution at *ɑ*/2 (for a confidence level of 95%, *ɑ* is 0.05 and the critical value is 1.96), “*Zβ*” is the critical value of the normal distribution at *β* (power of 80%, *β* is 0.2 and the critical value is 0.84), “*σ*2 ” is population variance, and “*d*” is the difference you would like to detect.

*N* = 25 each group

The study was performed in conformity with the Helsinki declaration and the protocol was approved by the local central ethics committee on human research (Ref. No DMIMS [DU]/IEC/2020-2021/51). The patients were explained in detail about the study protocol and written consent was obtained from each before inclusion in the study. A detailed history was recorded, and the patients were subjected to routine haematological and serological tests.

To be included in the study sample, the patients should be aged between 18 and 50 years with ASA Grade I status, BMI < 25 kg/m^2^, and have bifocal mandibular fractures intended for open treatment using mini plates under general anesthesia. Systemically compromised patients, comminuted and externally compound mandibular fractures, presence of pan facial fractures, patients with head and long bone injuries, lactating and nursing mothers and patients having bleeding disorders, impaired cognitive function, allergies to NSAIDs, peptic ulcer, bronchial asthma, alcohol, and substance abuse were excluded from the study.

A 1:1 randomized allocation was done into two groups using a computer-generated table of random numbers using a sealed opaque envelope, irrespective of their age, sex, and site of fractures. Fractures in the dentate region of the mandible were accessed transmucosally, while those involving the ramus and condyle were addressed transcutaneously. A single independent observer was assigned to ensure randomization and record the patient data. All the cases were operated by a single senior surgeon having considerable experience in maxillofacial trauma under general anesthesia. The operating surgeon, independent evaluator, and the patients were masked to the randomization making the study triple-blind in design.

Group K (study group) received a ketoprofen transdermal patch (100 mg Artho-Touch®, Sandor) and while group D served as control and received transdermal diclofenac sodium patches (100 mg; Zuventus® Healthcare, Ltd., Maharashtra, India). These were applied to non-hair-bearing parts of the body (Deltoid region, chest, upper back region, thigh) an hour prior to induction of anesthesia according to the randomization protocol by an independent researcher. The same researcher ensured consistency of the anesthetic and analgesic protocols. The anesthesia protocol included the premedication cocktail (pantoprazole, glycopylorrate, midazolam, butorphanol), induction using propofol, intubation under succinylcholine, and maintenance (nitrous oxide, isoflourane, and vecuronium bromide) and reversal (ondansetron, neostigmine, and glycopylorrate). No analgesics were given in the immediate PO phase.

In the immediate PO phase, pain severity was recorded using ‘the 10-point’ Visual Analogue Scale (VAS)[12]. Patients were asked to score the intensity of pain at 4, 6, 12, and 24 hourly. The duration of post-operative analgesia was calculated till the consumption of the first rescue analgesic. Tramadol hydrochloride 2 mg/kg was used as a rescue analgesic whenever the patient scored pain (VAS ≥ 4). The time to consumption of rescue analgesic, VAS score, and other outcomes, such as any allergic adverse responses, were documented depending on the information provided by the subjects. The point of consumption of rescue analgesics was considered the endpoint of the study.

Statistical analysis was conducted using descriptive and inferential statistics, including the chi-square test, unpaired t-test, the student’s paired test, and Statistical Package for Social Sciences (SPSS) 27.0 (IBM SPSS, Armonk, NY), and GraphPad Prism 7.0 version software (GraphPad Software, Inc., La Jolla, CA). Statistical significance was set at p<0.05.

## Results

The present study sample included 50 male patients (Table 1) with no dropouts from the study.

**Table 1:** Demographic details of the study population

N=50	Group-K (N=25)	Group-D (N=25)	P-value
Age	30.72 ± 4.24	31.74 ± 4.12	0.349, NS
Weight	49.8 ± 3.7	50 ± 3.5	0.12, NS
Height	160.0 ± 4.9	159.0 ± 3.1	0.14, NS
Body mass index	20.5 ± 3.7	21.4 ± 2.5	0.91 NS

The mean age range between (mean ± SD) 18-50 years was found to be 30.72 ± 4.24 for group K and 31.74 ± 4.12 for group D, with insignificant differences in weight, height, and body mass index among both groups K and D, respectively (P > 0.05). The mean duration of post-operative analgesia among patients in group D was 12.80 ± 0.57 and in group K it was 17.50 ± 0.78, which was found to be significant (P < 0.05) (Table 2).

**Table 2:** Comparison of duration of postoperative analgesia in two groups

	Group K	Group D	z-value
Duration of postoperative analgesia	17.50 ± 0.78	12.80 ± 0.57	4.71, p=0.007, S

The mean VAS score among patients of group K (2.35 ± 0.31) and group D (3.15 ± 0.41) was found to be statistically significant (p= 0.007) (Table 3).

**Table 3:** Mean Visual Analog Scale score among both groups

	Group K	Group D	z-value
Mean VAS score	2.35 ± 0.31	3.15 ± 0.41	0.8, p=0.007, S

The consumption of rescue analgesics in the first 24 hours of PO was observed after the completion of 12 hours; 48% (n=12) patients in group D required compared to 25% (n=5) in group K with a statistically significant (p = 0.007) difference (Table 4).

**Table 4:** Comparison of consumption of rescue analgesic in two groups

	Group K N=7	Group D N=12	z-value
No of rescue analgesic	1.60 ± 1.71	3.60 ± 1.17	3.04, p=0.007, S

## Discussion

Post-operative analgesia is one of the critical tenets of assessing the overall success of treatment and patient satisfaction and demands special attention. There is a lack of consensus regarding procedure-specific pain management protocols in oral and maxillofacial surgery. Essentially, pain occurs due to central and peripheral sensitization by inflammatory mediators triggered by a noxious stimulus. Pre-emptive analgesia intends to intercept central as well as peripheral sensitization, thereby moderating the amplification of PO pain [12].

The quest for an analgesic drug and route which can provide optimal analgesia with minimal adverse effects is ongoing. Recently, TDDS has emerged as a safe modality for post-operative analgesia and offers some unique advantages such as self-administration, non-invasive, effective, continual drug delivery, bypasses first-pass metabolism, greater bioavailability, helps to avoid gastrointestinal effects and other adverse effects (low plasma concentrations), trypanophobia, and enhanced patient compliance [8,10]. On application over the skin, the drug is absorbed transdermally down the concentration gradient into the systemic circulation continually and steadily, allowing drug concentrations to exert optimal therapeutic effects without any systemic adverse effects. Diwan et al. [13] found TDDS diclofenac to have comparable analgesic efficacy and fewer adverse effects when compared with the oral route, whilst others [14,15] have demonstrated the greater potency and safety of TDDS diclofenac over systemic routes unequivocally with better patient compliance [14-17]. Recently, in comparative trials, Kumar et al. and Metry et al. validated the efficacy of ketoprofen TDDS during venous cannulation [18-20].

Hence, the present study was instituted to evaluate the preemptive analgesic efficacy of diclofenac and ketoprofen TDDS in post-operative pain management following open treatment of bifocal mandibular fractures. The primary outcomes evaluated were the pain severity through a mean VAS score, the duration of analgesia, and the number of rescue analgesics required in the first 24 hours. Additionally, any local and systemic adverse effects were also noted.

The time to the onset of a formulation is largely manufacturer-dependent. With TDDS, this time varies from three to four hours to reach the ceiling effect in 10 to 12 hours with a 24-hour duration of action [21]. In the present study, the TDDS patch was applied before intubation so that the onset of action of the drug would start before the occurrence of the first peak of pain, thus exhibiting its pre-emptive effect.

The results of the present study have demonstrated that ketoprofen TDDS significantly provides profound analgesia compared with diclofenac TDDS post-operatively. The post-operative analgesia was measured using a 10-point VAS following surgery at 4, 6, and 12 hours post-operatively. The mean duration of post-operative analgesia was measured from the point of extubation till the consumption of the first rescue analgesic. The mean duration of post-operative analgesia among patients in group D was 12.80 ± 0.57 and in group K it was 17.50 ± 0.78 with a significant difference (P=0.0001*). The mean VAS score in the immediate post-operative period varied significantly with fewer scores in group K at 2, 4, 6, 8, 12, and 24 hours (p < 0.05) with only 25% (n=5) of patients requiring rescue analgesics. This difference in the duration of post-operative analgesia can be attributed to its low molecular weight (260 Da), which facilitates absorption and central desensitization by inhibiting the spinal cord nociceptor reflex activity of ketoprofen. This mechanism of ketoprofen is similar to opioids without the risk of respiratory depression. Therefore, ketoprofen is commonly indicated in musculoskeletal and joint disorders such as ankle injury, rheumatoid arthritis, osteoarthritis, and periarticular disease. Danish Khan et al. conducted a study to evaluate the effectiveness and acceptability of a transdermal patch of ketoprofen compared to a diclofenac patch for post-operative analgesia in patients having orthopaedic hip fracture operations and found ketoprofen to be more effective [1].

Similar results were observed by Bhargava et al. [2] and Shankar et al. [20]. The latter evaluated the effectiveness of a single-dose transdermal patch of ketoprofen compared to that of diclofenac in orthodontic patients after therapeutic excision of first premolar teeth. Patients who got a single-dose ketoprofen patch reported reduced post-operative pain and did not require rescue medication as compared to patients who underwent a diclofenac patch and had considerably greater pain in the first 24 hours. Jadhav et al. conducted a prospective, double-blind, randomized study to compare the pre-emptive analgesic efficacy of diclofenac and ketoprofen transdermal patches in the management of immediate post-operative pain following bi-jaw surgery and concluded that ketoprofen had the edge over the diclofenac transdermal patch with respect to analgesic efficacy [8].

The need for additional analgesia in the first 24 hours of PO was observed after the completion of six hours; 24% (n=12) of patients in group D required compared to 14% (n=7) in group K with a statistically significant (p-value=0.007) difference. The results indicate the potency of ketoprofen over diclofenac. Similar results were observed by Velásquez et al. [21]. Ketoprofen has an established efficacy over ibuprofen and diclofenac in sports injuries, orthopaedic and rheumatic pain [22]. In the present study, none of the patients experienced adverse effects or allergic reactions with the application of TDDS.

Diclofenac sodium is a widely prescribed analgesic for post-operative pain control. It has been concluded that the analgesic efficacy of a single-dose TDDS diclofenac patch is comparable to intra-muscular and oral routes [7]. On the contrary, Bachalli et al. found TDDS diclofenac ineffective in providing adequate analgesia in the immediate PO period. The authors recommend oral diclofenac followed by TDDS diclofenac for effective pain control [9]. Upon comparison with TDDS ketoprofen, the latter was found to be efficacious with fewer VAS scores and fewer analgesic requirements in orthopaedic surgeries involving the lower limb [23]. The results are in agreement with the observations of the present study in terms of lower PO mean VAS scores and analgesic requirements. Similar results were also observed by Jadhav et al. and Bhargava et al. [2,8]. TDDS provided a continual absorption of the drug across the dermis, thus allowing for consistent serum drug levels with increased tissue concentration and low plasma concentration [24]. The present study demonstrated the superior analgesic efficacy of ketoprofen over diclofenac transdermal patches.

This is the first study of its kind to evaluate the efficacy of ketoprofen and diclofenac sodium TDDS in mandibular fractures. The limitations of the present study included the limited sample size and the variability of pain perception between two individuals. Furthermore, the present study did not take into consideration quality of life issues. Further multi-centric clinical trials are needed to endorse TDDS as the sole method to achieve post-operative analgesia.

## Conclusions

There is a dearth of literature showing the superiority of the ketoprofen transdermal patch over diclofenac patches in the maxillofacial region. The present study showed the superiority of the Ketoprofen transdermal patch over the diclofenac patch in terms of providing adequate post-operative analgesia for a prolonged duration, less requirement for post-operative rescue analgesics, and minimal adverse events. This study has the potential to overlay the usage of conventional diclofenac patches in reducing pain post-operatively following maxillofacial trauma.
